# Seroprevalence of human enterovirus A71 in Guangzhou, China, 2019–2021

**DOI:** 10.1016/j.bsheal.2023.05.002

**Published:** 2023-05-06

**Authors:** Huimin Lian, Huimin Jiang, Lina Yi, Jing Sun, Huaping Xie, Ming Qiu, Limei Sun, Huifang Lin, Mingda Yang, Lin Qu, Haiyi Yang, Jing Lu, Hanri Zeng

**Affiliations:** a School of Public Health, Southern Medical University, Guangzhou 510515, China; b Guangdong Provincial Institution of Public Health, Guangdong Provincial Center for Disease Control and Prevention, Guangzhou 511430, China; c Guangdong Workstation for Emerging Infectious Disease Control and Prevention, Guangdong Provincial Key Laboratory of Pathogen Detection for Emerging Infectious Disease Response, Guangdong Provincial Center for Disease Control and Prevention, Guangzhou 511430, China; d Health Commission of Heping District, Shenyang City, Liaoning 110003, China; e Guangzhou Center for Disease Control and Prevention, Guangzhou 510440, China; f School of Public Health, Sun Yat-Sen University, Guangzhou 510080, China; g School of Public Health, Jinan University, Guangzhou 510632, China

**Keywords:** Enterovirus A71, Vaccine, Seroepidemiology

## Abstract

Enterovirus A71 (EV-A71) is a significant hand-foot-mouth disease (HFMD) etiology. The inactivated EV-A71 vaccines were approved in China in 2016. However, the seroprevalence of EV-A71 after the vaccine application and its potential association with the EV-A71 epidemic in the population are rarely studied. In this study, we analyzed the incidence of EV-A71 infection and seroepidemiology in Guangzhou City, China. From 2019 to 2021, 167,920 clinically confirmed HFMD cases were reported in Guangzhou. In 6,868 enterovirus-positive samples, Coxsackievirus A6 and Coxsackievirus A16 were dominant genotypes, and only 3 EV-A71-positive samples were detected, highlighting the deficient epidemic activity of EV-A71. Microneutralization assay was performed on 1,000 representative serum samples. Notably, the seroprevalence and geometric mean titer (GMT) decreased significantly in 2020, and that in the < 3-year age group were increased and even higher than that in 3–5-year age group in 2019 and 2021, which was contrary to our previous surveillance result and other studies in Guangzhou. Furthermore, a moderate decline of GMT level was observed following the vaccination, but the seropositive serums were still detected for 49 months after second immunization, suggesting the long-term persistence of the immunity. Our seroepidemiology study revealed relatively higher neutralizing antibody activity in the susceptible population after the EV-A71 vaccine was adopted in 2016 in Guangzhou. It may be one of the reasons for the lower epidemic activity of EV-A71 in Guangzhou from 2019 to 2021.

## Highlights

• EV-A71 as a major agent of HFMD was rarely detected after 2019 in Guangzhou, China.
• The increased seroprevalence and nAb titer was observed after vaccine adoption.
• The enhanced immunity in susceptible populations may lower viral epidemic activity.

## 1 Introduction

Hand-foot-mouth disease (HFMD) is an acute contagious disease primarily affecting infants and children younger than five years [1], [2]. Since 1997, HFMD has caused a significant burden in the Asia-Pacific region [3]. For example, a large outbreak of HFMD occurred in mainland China in 2008. Since then, HFMD ranked first in “Category C Notifiable Infectious Diseases”, and 4,034,733 HFMD cases were reported in China from 2019 to 2021.

A wide variety of enteroviruses causes HFMD. Enterovirus A71 (EV-A71) has been identified as one of the dominant enterovirus genotypes for the HFMD epidemic in mainland China since 2008 [4]. More importantly, it is regarded as the predominant causative pathogen of severe or fatal complications [2], [5]. The periodic circulation of EV-A71 and other enterovirus genotypes like Coxsackievirus A6 (CVA6), Coxsackievirus A16 (CVA16), and Coxsackievirus A10 (CVA10) was regarded as the reason for the continuous HFMD epidemic in the mainland of China in the last 14 years [6], [7], [8], [9]. In Guangdong Province, China, EV-A71 was the primary pathogen causing the outbreaks of HFMD from 2008 to 2012. After that, the co-circulation of CVA6, CVA10, CVA16, and EV-A71 was observed, with different genotypes detected as primary causative agents each year [9], [10].

There are no specific effective drugs or therapies to treat HFMD cases. The inactivated EV-A71 vaccines (subgenotype C4) were developed and licensed in mainland China in 2016 [11]. The clinical trial results showed that the inactivated EV-A71 vaccine has remarkable efficiency and safety in protecting children aged 6–59 months from EV-A71 infection [12], [13], [14]. However, the data from the real world are rare. Guangzhou City is one of the most severe cities in HFMD with high morbidity, and the HFMD epidemic pattern of the pathogen is changing [7], [9], [10].

To fill the existing gaps in knowledge about the seroprevalence of EV-A71 after the vaccine application and its potential association with the EV-A71 epidemic, we conducted a cross-section seroepidemiological study for neutralizing antibodies against EV-A71 in different age groups in Guangzhou City, 2019–2021.

## 2 Materials and methods

### 2.1 The serum samples

The EV-A71 seroprevalence surveillance is a part of the Enhanced Surveillance Program of Immune Status of the Guangdong Population. All serum samples used in this study were collected from a sentinel hospital in Panyu district in Guangzhou from June to October. The serum samples were stored at −80 °C until testing. A total of 1,000 serum samples collected from 2019 to 2021 were classified into five age groups (< 3, 3 ∼ < 5, 5 ∼ < 25, 25 ∼ < 60, and ≥ 60 years). The demographic profile of the participants enrolled in this study is illustrated in Table 1. The Guangdong Provincial Center for Disease Control and Prevention ethics committee approved the surveillance program.

**Table 1:** Demographic characteristics of subjects in the seroepidemiological survey.

Year	Age (years)					Total
	< 3	3 ∼ < 5	5 ∼ < 25	25 ∼ < 60	≥ 60	
2019	73	35	43	49	–	200
2020	63	89	81	86	81	400
2021	80	80	80	80	80	400
Total	216	204	204	215	161	1,000

All serum samples used in this study were collected from individuals who had participated in Enhanced Surveillance Program of Immune Status of Guangdong Population.

### 2.2 Neutralizing antibody assay

The microneutralization assay was performed as previously described [15], [16]. EV-A71/Guangdong/405/2019 (C4a genotype) strain was used to quantify neutralizing antibody levels to EV-A71. Firstly, serum samples were inactivated at 56 °C for 30 min, diluted from 1:8 to 1:1,024, and then incubated at 37 °C for 1 h with an equal volume of 100 TCID_50_ (100 half tissue culture infective dose) of EV-A71. Next, we transferred them into 96-well plates precoated with rhabdomyosarcoma (RD) cells (2 × 10^5^ cells/mL). Finally, these plates were incubated in a 5% carbon dioxide incubator at 37 °C for seven days. Cell control, serum control, and virus control were included in each plate. Viral back titration was conducted in each test. Cytopathic effect (CPE) was observed with an inverted microscope daily. The neutralizing antibody titer of the sample was defined as the highest plasma dilution that could prevent CPE development in 50% of the virus-infected wells. A neutralizing antibody titer of ≥1:8 was considered a threshold for positivity.

### 2.3 Statistical analysis

Statistical analyses were performed with SPSS version 13.0 software (SPSS Inc., Chicago, IL, USA). The seroprevalence and GMT with 95% confidence intervals (CI) were calculated. The chi-squared, Kruskal–Wallis, or Mann–Whitney tests were used to test the significant differences in seroprevalence or GMT between groups, and considered *P* < 0.05 statistically significant. The neutralizing antibody titers > 1:1,024 were assigned 1:1,024, and < 1:8 were assigned 1:4 [4], [17], [18], [19].

## 3 Results

### 3.1 HFMD cases and enterovirus genotype distribution in Guangzhou, 2019–2021

167,920 clinically confirmed HFMD cases were reported in Guangzhou from 2019 to 2021, and a significant decrease appeared in 2020. 10,036 samples were sent to Guangdong Provincial Center for Disease Control and Prevention for verification and further genotype classification. As illustrated in Table 2, 6,868 samples were detected as enterovirus positive. The dominant genotypes were CVA6 (n = 2,573, 81.2%) and CVA16 (n = 1,264, 39.9%); only one EV-A71 positive sample was detected each year, highlighting the shallow epidemic activity of EV-A71 from 2019 to 2021.

**Table 2:** EV serotypes of clinically confirmed HFMD cases in Guangzhou City, China in 2019–2021.

Year	Number of cases	Amount of test	EV serotypes (number of enterovirus-positive cases)					
			Total	EV-A71	CVA16	CVA6	CVA16 + CVA6	other EVs
2019	92,195	3,999	2,856	1	560	1,289	18	988
2020	13,138	1,598	778	1	27	504	2	244
2021	62,587	4,439	3,234	1	677	780	17	1,759
Total	167,920	10,036	6,868	3	1,264	2,573	37	2,991

The above data was collected from Guangdong Provincial Center for Disease Control and Prevention.

Abbreviations: HFMD, hand-foot-mouth disease; EV, enterovirus; EV-A71, Enterovirus A71; CVA 16, Coxsackievirus A16; CVA6, Coxsackievirus A6.

### 3.2 Seroprevalence and GMT level of EV-A71 in the Guangzhou population

**Fig. 1:**
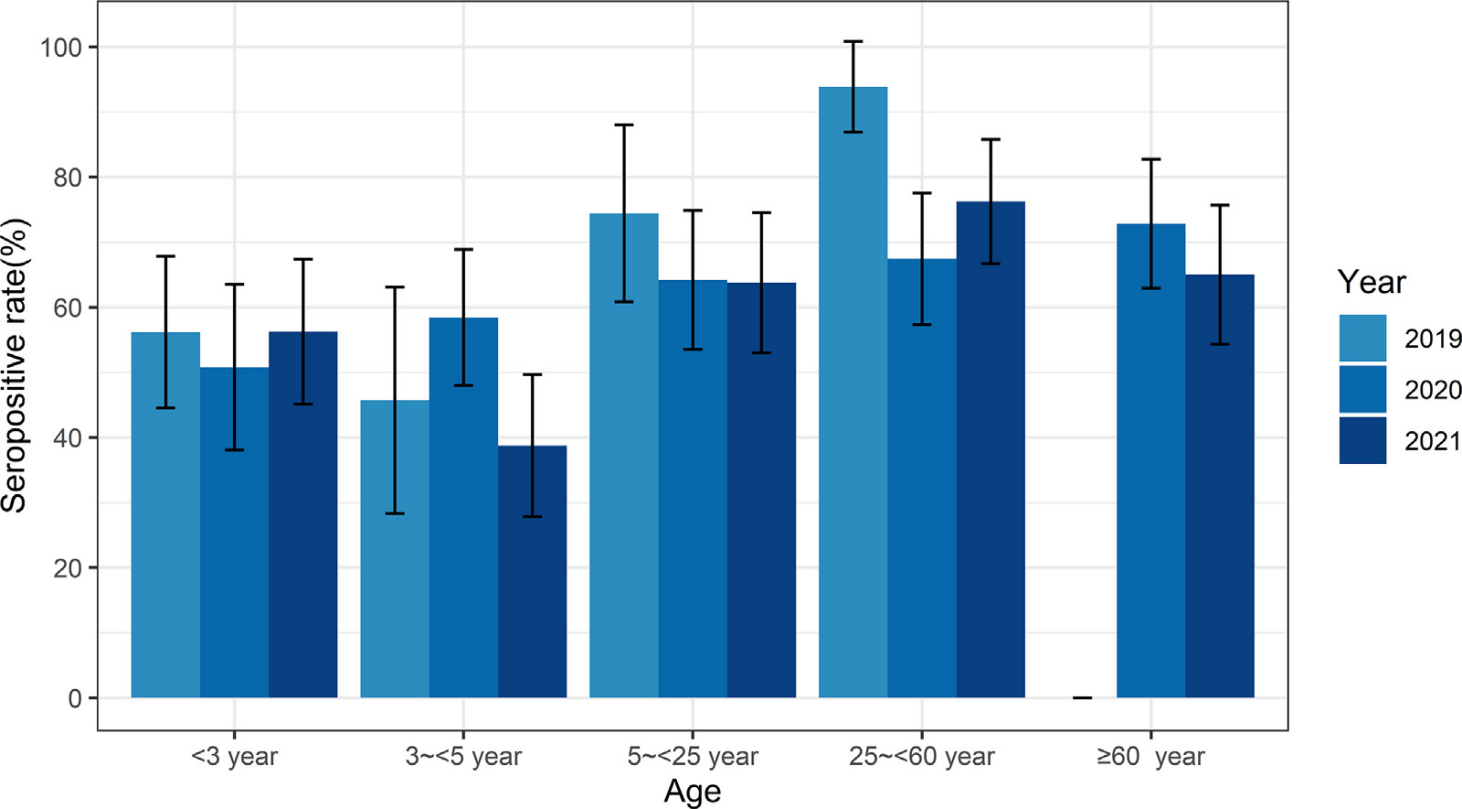
Seroprevalence of EV-A71 neutralizing antibodies in different age groups in Guangzhou City, China from 2019 to 2021. Abbreviation: EV, enterovirus.

Serum samples were collected from healthy individuals, and specific neutralizing antibody levels against EV-A71 were tested by microneutralization assay. The samples were tested by year: 200, 400, and 400 samples were tested in 2019, 2020, and 2021, respectively. As shown in Fig. 1, the overall seroprevalences of EV-A71 ranged from 45.71% to 93.88% in 2019, 50.79% to 72.84% in 2020, and 38.75% to 76.25% in 2021 (*P* > 0.05). As expected, the seroprevalences were varied in the different age groups, similar to our and other previous studies [20], [21]. Notably, the relatively high seroprevalences were detected in the < 3-year age group (56.16%, 95% CI: 44.51%–67.82%; 56.25%, 95% CI: 45.14%–67.36%) which were even higher than those aged 3–5 years (45.71%, 95% CI: 28.35%–63.08%; 38.75%, 95% CI: 27.84%–49.66%) in the year of 2019 and 2021, respectively. Again, this differed from our previous seroprevalence surveillance from 2007 to 2009, for which the seroprevalence in the < 3-year age group was the lowest.

**Fig. 2:**
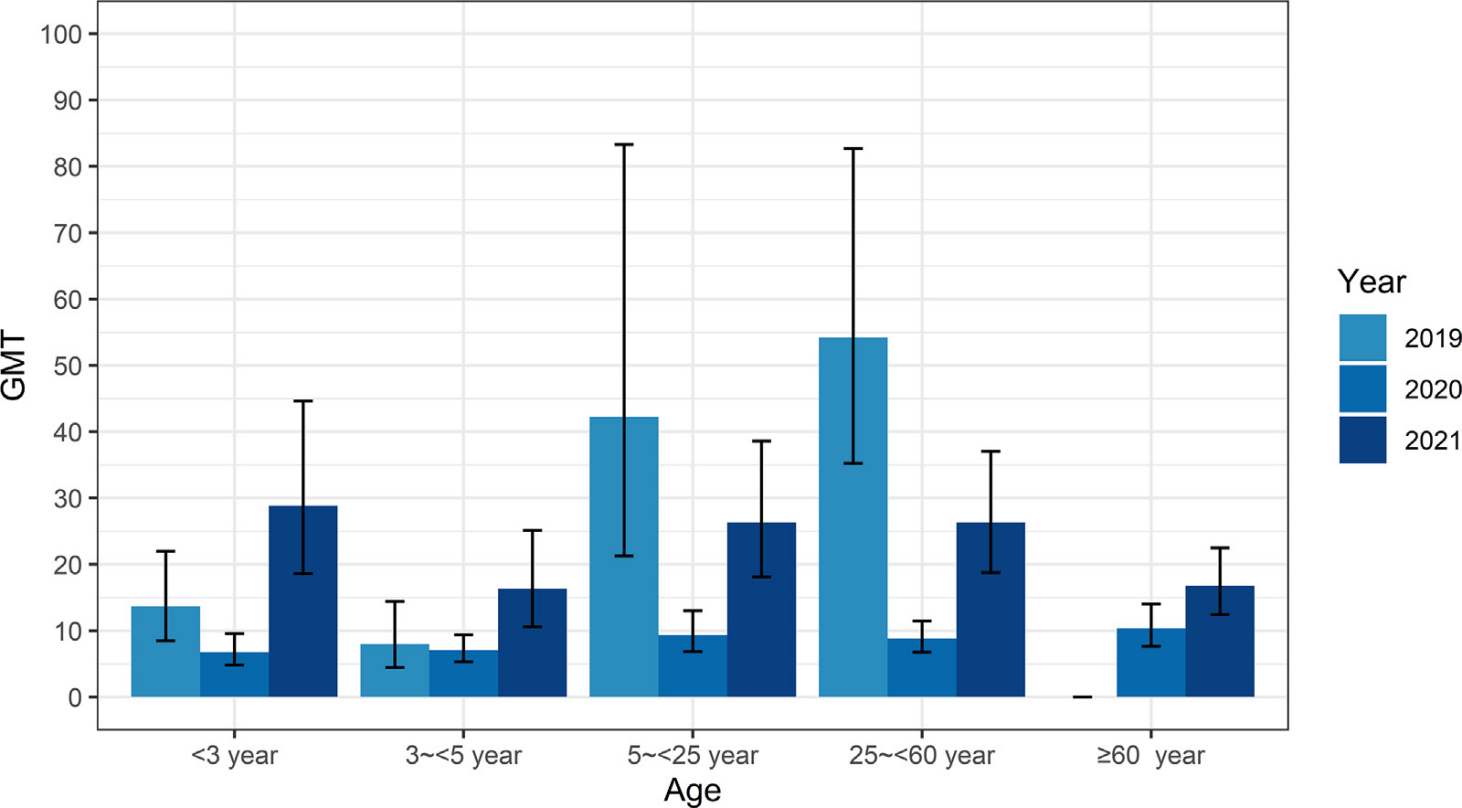
Geometric mean titer (GMT) of Enterovirus A71 (EV-A71) neutralizing antibodies in different age groups in Guangzhou City, China from 2019 to 2021.

For those seropositive samples, the overall GMT of neutralizing antibodies to EV-A71 in 2019 and 2021 were 22.16 (95% CI: 16.68–29.45) and 22.32 (95% CI:18.77–26.35), which were significantly higher than 2020 at 8.46 (95% CI:7.36–9.65) (*P* < 0.001). Consistent with the seroprevalence, the GMT of neutralizing antibodies to EV-A71 was increased with age except for those aged < 3 years and the population aged ≥ 60 years (Fig. 2). No significant gender-specific difference in seroprevalence and GMT of EV-A71 was observed (*P* > 0.05). Our results highlighted the increase of the seroprevalence and GMT of neutralizing antibodies to EV-A71 in the susceptible population in Guangzhou City from 2019 to 2021.

### 3.3 Immunogenicity

In 2021, all participants provided their vaccination information, and we can roughly evaluate neutralizing antibody levels after the vaccination over time. Among the subjects aged < 5 years, 80 had completed two doses of EV-A71 vaccination.

To compare the results with previous clinic trail data, we separated the collection-vaccination time spans into six groups: 1 (n = 3), 7 (n = 1), 13 (n = 14), 25 (n = 22), 37 (n = 35), and 49 months (n = 5) after second vaccination [11], [22]. A decrease in seroprevalence and neutralizing antibody titers was observed according to the time spans.

The seroprevalences of EV-A71 were 100.0% (95% CI: 100.0%–100.0%), 100.0% (95% CI: 100.0%–100.0%), 78.6% (95% CI: 54.0%–100.0%), 77.3% (95% CI: 58.3–96.3%), 57.1% (95% CI: 39.9%–74.4%) and 40.0% (95% CI: 0.0%–100.0%) in month 1, 7, 13, 25, 37 and 49, respectively (*P* = 0.005), indicating a slight decline of the neutralizing antibody against EV-A7 from the seventh month after the second injection.

**Fig. 3:**
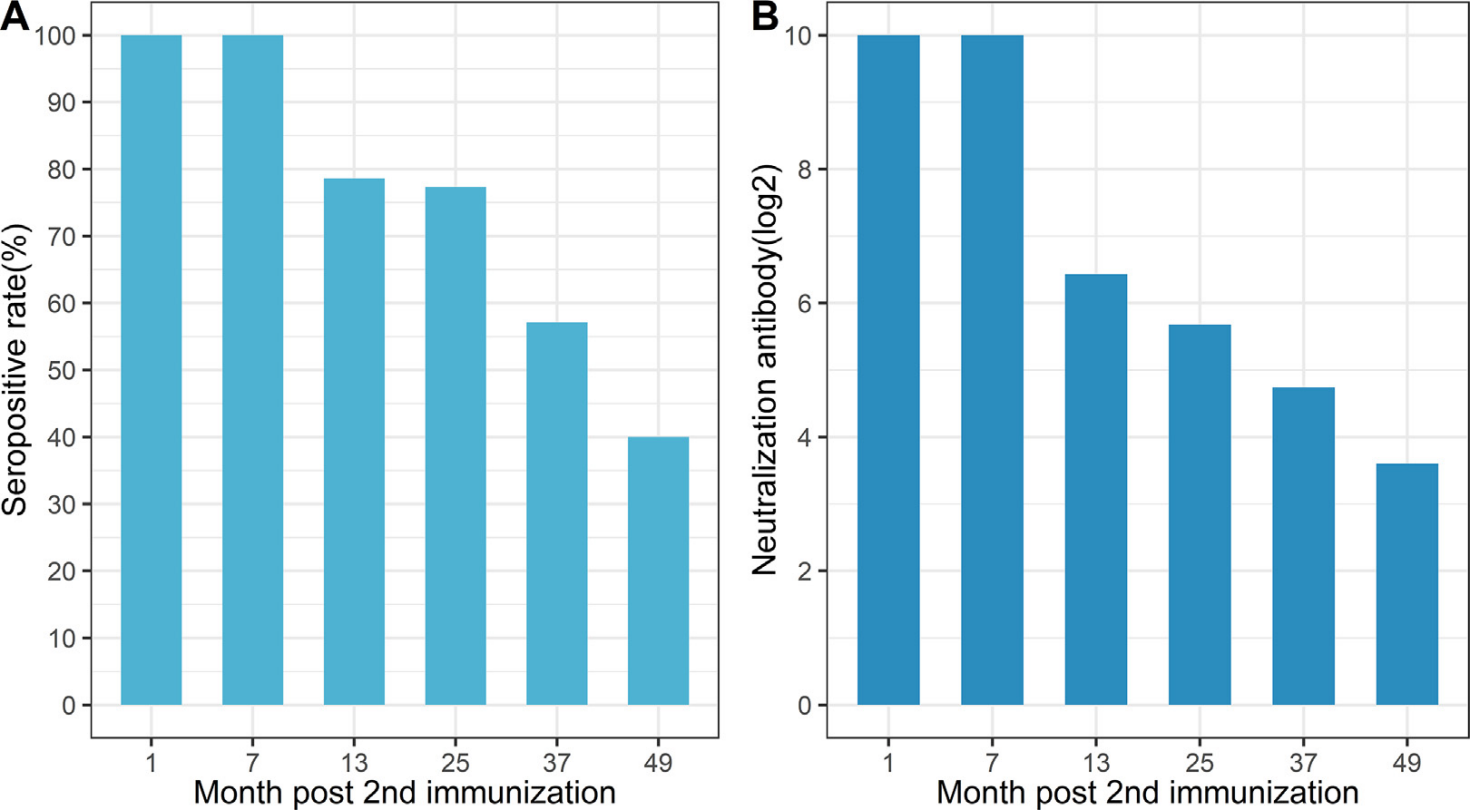
Geometric mean titer (log_2_) and seroprevalence of Enterovirus A71 (EV-A71) neutralizing antibodies at different time points after the second vaccination from Month 1 to 49. The geometric mean titers (log_2_) (A) and seroprevalence (B) of neutralizing antibodies to EV-A71 were shown above the bar.

The neutralizing antibody titers remained at a relatively high level with GMT of 1:1,024 on months 1 and 7; GMTs in months 13, 25, 37, and 49 were 86.16 (95% CI: 27.86–266.32), 51.34 (95% CI: 24.71–106,60), 26.78 (95% CI: 14.17–50.60) and 12.13 (95% CI: 1.67–87.97), respectively (*P* > 0.05), indicating the persistence of immunity following the EV-A71 vaccination (Fig. 3).

## 4 Discussion

The monovalent EV-A71 vaccine was introduced in mainland of China in 2016. Therefore, surveillance on seroepidemiology and the EV-A71 distribution in HFMD cases is needed. The seroprevalences in different age groups provide valuable information on the population's immunity to the virus and the disease susceptibility of different populations, which is essential for guiding future immunization programs. In this study, we conducted a cross-sectional seroepidemiological survey of 1,000 serum samples collected from different age groups in Guangzhou from 2019 to 2021 to gain a comprehensive insight into the seroprevalence of EV-A71. Our data showed that the neutralizing antibodies titer to EV-A71 in 2020 was significantly lower than that in 2019 and 2021, consistent with the incidence trend of HFMD. HFMD pathogens mainly spread through close contact with contaminated hands and personal products, and kindergartens are major places for HFMD outbreaks [23], [24], [25]. Therefore, kindergarten closure, handwashing, and wearing masks may effectively block the transmission of HFMD, possibly reducing the population's immune level.

Our surveillance results also showed an increase of seroprevalence and GMT to EV-A71 in the susceptible population (aged < 3 years) from 2019 to 2021 when compared to our and other surveillance results in Guangdong Province from 2007 to 2009 [26] or Guangzhou City from 2014 to 2015 [21]. The previous studies suggested that the seroprevalence and GMT of neutralizing antibody to EV-A71 increased with age, indicating that the elder group was significantly higher than the younger group [4], [19], [20], [21], [26]. Our results indicated increased population immunity to EV-A71 in the susceptible population following the vaccination program launched in 2016.

The EV-A71 vaccine remains the only available vaccine in China at present. However, evidence of EV-A71 vaccine effectiveness in the “real world” from the population-based study is still rare. The previous clinical trial data showed that inactivated EV-A71 vaccine-induced immunogenicity has persisted satisfactorily for five years [11], [22]. This study observed an overall decrease in the seroprevalence and GMT. However, a relatively high seroprevalence (100%) and neutralizing antibody titer (1:1,024) were detected seven months after the 2nd vaccination. In addition, 40% of serums were still seropositive 49 months after the second vaccination. These results indicating the inactivated EV-A71 vaccine could provide a good persistence of immune protection in the susceptible population [11], [27].

Despite the incidence of EV-A71 decreased, the number of HFMD cases remained substantial in mainland China due to the increasing ratio of the non-EV-A71 EVs (CVA6, CVA10, and CVA16) [10], [28], [29]. The successful application of the EV-A71 vaccine could be one of the reasons for the decline in EV-A71 prevalence [10]. Therefore, the development and application of multivalent vaccines should be discussed in the future. In addition, EV-A71 still was responsible for the most severe and fatal HFMD cases in mainland China. The genetic variation and antigenicity of EV-A71 should be continuously monitored, especially when the population immunity reaches a relatively high level.

## 5 Conclusions

In summary, our study provided an analysis of the incidence of EV-A71 infection and age-stratified seroprevalence from 2019 to 2021 in Guangzhou City. In contrast to the previous studies, the relatively higher neutralizing antibody to EV-A71 was observed in the < 3-year age group from 2019 to 2021, highlighting the persistence of immunity and the increase of immune activity level to EV-A71 in the susceptible population after the adoption of EV-A71 inactivated vaccine.

## Ethics statement

As a public health response to an emerging infectious disease outbreak, written informed consent could be waived. Data and serum sample collection procedures as part of the enhanced population immunity suveillance progam were approved by the Health Commission of the Guangdong Province and Guangdong Provincial Center for Disease Control and Prevention ethics committee.
